# The effects of specific vegetable subtypes on constipation incidence in the general United States population

**DOI:** 10.3389/fnut.2024.1403636

**Published:** 2024-07-24

**Authors:** Chenyu Jiang, Yaojian Shao

**Affiliations:** ^1^Department of Geriatric, Taizhou Central Hospital (Taizhou University Hospital), Taizhou, Zhejiang, China; ^2^Department of Gastroenterology, Taizhou Central Hospital (Taizhou University Hospital), Taizhou, Zhejiang, China

**Keywords:** tomatoes, potatoes, vegetables, constipation, FPED

## Abstract

**Background:**

While the intake of larger quantities of vegetables has been linked to a reduction in constipation risk, which vegetables in particular underlie this risk reduction remains incompletely understood. As such, the present study was developed to explore correlations between the intake of particular vegetable types and the risk of constipation.

**Methods:**

This cross-sectional analysis was based on data from the National Health and Nutrition Examination Survey (NHANES) collected from 2005-2010. Classifications and intake assessments for different vegetables were assessed with the Food Patterns Equivalents Database (FPED), while stool frequency or stool consistency was used to define constipation. Relationships between the intake of particular vegetable components and constipation were assessed through a weighted logistic regression approach. Subgroup and restricted cubic spline (RCS) regression analyses were further employed to explore associations between specific vegetable subtypes and constipation.

**Results:**

This study included 13,860 eligible subjects, of whom 1,405 and 12,455 were respectively classified into the constipated and non-constipated groups. Following multivariate adjustment, the intake of non-starchy vegetables including orange, red, dark green, and other vegetables was found to be positively associated with a reduction in constipation risk. In contrast, constipation was unrelated to total starchy vegetable or potato intake. Tomatoes, in particular, were associated with a marked decrease in constipation risk (odds ratios: 0.80, 95% confidence interval: 0.71–0.91). These results were confirmed through RCS and subgroup analyses.

**Conclusion:**

Non-starchy vegetables, particularly tomatoes, were found to be associated with a pronounced reduction in constipation risk, which was unaffected by the intake of potatoes or starchy vegetables.

## 1 Introduction

Constipation is among the most prevalent gastrointestinal disorders, adversely impacting the quality of life of individuals throughout the world (1). The global prevalence of constipation is estimated at 10.1% worldwide among adults when adopting the stricter Rome IV criteria, with women more often being affected as compared to men (2). Constipation is a complex, multifactorial condition that is shaped by factors including diet, lifestyle, drug intake, depression, and socioeconomic status (3). Of these factors, both lifestyle and dietary composition are modifiable risk factors (4). The relationship between dietary components and the incidence of constipation has been the subject of particularly high levels of interest, such that dietary management is widely employed as a key facet of any clinical effort aimed at treating constipation (5).

Vegetables are an essential component of the human diet, and they are also generally believed to be closely tied to the incidence of constipation (6). High levels of vegetable intake have been reported to lower the risk of constipation. This is consistent with the fact that many vegetables are rich in flavonoids, trace elements, vitamins, and dietary fiber (7). Soluble dietary fiber is well known to help alleviate constipation, with pectin, which is a form of soluble fiber, offering clear short- and long-term efficacy in patients suffering from constipation as the result of slow gastrointestinal transit (8). Trace elements including selenium, magnesium, and phosphorus have also been found to be negatively correlated with constipation (9–11). Most research to date has focused on exploring relationships between constipation and particular dietary patterns or the intake of vegetables (12). The vegetable-rich Mediterranean diet, for example, can reportedly significantly improve bowel movements (13–15). Current understanding of the correlative relationships between particular vegetable categories and constipation risk, however, remains limited. Under current dietary guidelines, all vegetables are regarded equally. In reality, different vegetables have varied nutritional content; accordingly, the Food Patterns Equivalents Database (FPED) categorizes them into four groups, including dark green, orange/red, starchy, and other. The diverse nutrient components in different types of vegetables lead to discrepant associations with certain diseases and the risk of mortality (16, 17). There is thus a pressing need to fully explore the potential distinct effects that these different vegetable subtypes may have on the incidence of constipation.

In an effort to address this knowledge gap, the present study leveraged data from the NHANES study to conduct a comprehensive analysis of the association between particular categories of vegetables and the risk of constipation among adults in the United States.

## 2 Methods

### 2.1 Study subjects

The NHANES study is an ongoing effort to collect representative samples of the United States population every other year through a complex and comprehensive approach in order to better understand the nutritional and health status of the United States population. The National Center for Health Statistics Ethics Review Board provided approval for the NHANES study, and all participants provided written informed consent. For the present study, data were collected from NHANES participants from 2005 to 2010 as these individuals had accessible bowel health questionnaire (BHQ) data, which is a standardized assessment tool designed to evaluate an individual’s bowel habits, symptoms, and overall gastrointestinal health. Participants eligible for this analysis were adults 20+ years of age who had a complete BHQ and at least one valid 24-h dietary recall entry. Of the 31,034 NHANES participants from 2005 to 2010, 14,348 met these criteria. Of these individuals, 407 who were pregnant and 81 who were taking laxatives were excluded, while the remaining 13,860 comprised the study population.

### 2.2 Vegetable intake assessment

Dietary information was attained from the participants completed at least one valid 24-h dietary recall interviews. The FPED was used to classify and assess the intake of different types of vegetables by study participants. Vegetables or vegetable juices were grouped into four categories: dark green, orange/red, starchy, and other. As beans and peas (legumes) can be treated as protein foods, they were not categorized in the vegetable group but were included as covariates in the present analyses. Extracted vegetable types for this study included dark green vegetables, red and orange vegetables (tomatoes, other red and orange, and total red and orange), starchy vegetables (potatoes, other starchy vegetables, and total starchy vegetables), other vegetables, and total vegetable intake (not including legumes). Dark green vegetables included arugula, Chinese cabbage (bak choy), and mustard greens, among others. Red and orange vegetables included tomatoes, carrots, and red chili peppers, among others. Starchy vegetables included potatoes, corn, green peas, yam, and others. Other vegetables included bean sprouts, beets, bitter melon, and others. Tomatoes and potatoes were also extracted separately in an effort to better understand their relationships with constipation status. For further details regarding vegetable categories and cup equivalent weights, see the FPED database Methodology and User Guide.

### 2.3 Definition of constipation

Data from the NHANES database pertaining to stool frequency or consistency, which were recorded for 30 days prior to data collection by participants, were utilized to define constipation. The Bristol stool form scale (BSFS) was used to define stool consistency based on detailed descriptions and corresponding colored cards for seven possible stool types. Participants were directed to respond to the following question: “Please look at this card and tell me the number that corresponds with your usual or most common stool type?” Constipation was defined by those participants whose “usual or most common” stool type was either BSFS type 1 (separate hard lumps) or type 2 (lumpy, sausage-like), whereas BSFS types 3–7 were indicative of the absence of constipation. Stool frequency was assessed based on participant responses to the question “How many times per week do you usually have a bowel movement?” Constipation was defined by a response of fewer than three times per week, whereas 3+ times per week were indicative of the absence of constipation. Participants who satisfied either of the two conditions were clinically diagnosed with constipation (14).

### 2.4 Study covariates

Other participant information from the NHANES database that was assessed in the present study included age (years), sex (male, female), ethnicity (Mexican American, Other Hispanic, Non-Hispanic White, Non-Hispanic Black, and other), family poverty to income ratio (PIR), educational status (< high school, high school, > high school), body mass index (BMI, kg/m^2^), energy intake (kcal), total fat intake (g), total carbohydrate intake (g), total fruit intake (cup eq.), legumes intake (cup eq.), and whether or not patients had a history of smoking [no (<100 cigarettes lifetime or > 100 cigarettes lifetime and smoke not at all now), yes (>100 cigarettes lifetime and smoke some days or everyday)], alcohol intake [no (<12 lifetime drinks or 12+ drinks per year but none in the last year), yes (drinking over the past 12 months)], depression, diabetes, hypertension, and recreational activity [(for NHANES 2007–2010) or muscle strength (for NHANES 2005–2006)], work activity [(for NHANES 2007–2010) or tasks home/yard (for NHANES 2005–2006)], and active commuting (walk/bicycle). Diabetes mellitus was defined according to self-reported diagnoses, the use of insulin or other antidiabetic agents, fasting blood glucose levels ≥126 mg/dL, HbA1c levels ≥6.5%, or serum glucose ≥200 mg/dL at 2 h after a 75 g oral glucose load. Hypertension was defined by systolic and/or diastolic blood pressure values ≥140 and ≥ 90 mmHg, respectively, self-reported diagnoses, or the use of antihypertensive medications. Depression was defined based on responses to the 9-item Patient Health Questionnaire 9 (PHQ-9), which is validated and publically available, with a score ≥ 10 being indicative of depression.

### 2.5 Statistical analyses

To control for the complex multi-stage cluster design of the underlying data, appropriate NHANES sample weights were applied. Categorical data were given as numbers (weighted percentages) and compared with chi-squared tests, whereas continuous variables were given as means ± standard error (SE) and compared using Student’s *t*-tests. Odds ratios (ORs) and 95% confidence intervals (CIs) were computed with univariate and multivariate-adjusted logistic regression analyses when assessing associations between the intake of particular vegetable components and constipation. Model 1 was unadjusted, whereas Model 2 was adjusted to account for age, sex, and ethnicity, and Model 3 was further adjusted for education, BMI, energy intake, total fat intake, total carbohydrate intake, total fruit intake, legumes intake, smoking, drinking, hypertension, depression, recreational activity, work activity, active commuting and diabetes mellitus. The potential nonlinearity of these statistical relationships was assessed using restricted cubic spline (RCS) models. Subgroup analyses were additionally conducted based upon sex, ethnicity, education, hypertension, smoking, drinking, recreational activity, work activity, active commuting, diabetes mellitus, and depression. R (v 4.2.2) was utilized to conduct all analyses, using *p* < 0.05 as the threshold when defining significance.

## 3 Results

### 3.1 Participant characteristics

This study included 13,860 adults from the United States, of whom 1,405 were classified as being affected by constipation. The characteristics of these subjects are presented in Table 1. Significant differences in most vegetable subtypes were identified when comparing participants with and without constipation, with the exception of potatoes and other starchy vegetables. Individuals in the constipation group were more likely to be female, younger, to have a lower PIR, to exhibit lower levels of energy, fat, carbohydrate, and fruit intake, to be non-drinkers, to have a lower BMI, to have a lower educational status, to exhibit reduced recreational activity and work activity, and to have depression.

**Table 1:** Participant characteristics.

	Non-constipation	Constipation	*p* value
	*N* = 12,455	*N* = 1,405	
Total vegetable (cup eq.)	3.13 (0.04)	2.51 (0.07)	< 0.0001
Dark green vegetable (cup eq.)	0.28 (0.01)	0.20 (0.02)	< 0.001
Total red and orange vegetable (cup eq.)	0.80 (0.02)	0.61 (0.03)	< 0.0001
Tomato (cup eq.)	0.63 (0.01)	0.47 (0.02)	< 0.0001
Other red and orange vegetable (cup eq.)	0.18 (0.01)	0.14 (0.01)	0.01
Total starchy vegetable (cup eq.)	0.89 (0.02)	0.81 (0.04)	0.04
Potato (cup eq.)	0.71 (0.02)	0.65 (0.04)	0.1
Other starchy vegetable (cup eq.)	0.18 (0.01)	0.16 (0.01)	0.14
Other vegetable (cup eq.)	1.16 (0.02)	0.89 (0.03)	< 0.0001
Age (years)	47.23 (0.36)	45.30 (0.59)	0.002
PIR	3.13 (0.04)	2.60 (0.07)	< 0.0001
Body mass index (BMI, kg/m^2^)	28.79 (0.12)	27.75 (0.26)	< 0.001
Energy intake (kcal)	2191.03 (17.00)	1899.86 (33.50)	< 0.0001
Total fruit (cup eq.)	1.96 (0.04)	1.62 (0.05)	< 0.0001
Legumes (cup eq.)	0.21 (0.01)	0.18 (0.02)	0.14
Total fat intake (g)	83.16 (0.87)	71.02 (1.72)	< 0.0001
Total carbohydrate(g)	261.68 (1.91)	238.76 (3.96)	< 0.0001
Sex (%)			< 0.0001
Female	5,872 (48.46)	972 (74.29)	
Male	6,583 (51.54)	433 (25.71)	
Race (%)			< 0.0001
Mexican American	2,260 (7.86)	226 (8.29)	
Non-Hispanic Black	2,394 (10.35)	368 (16.57)	
Non-Hispanic White	6,263 (72.56)	619 (64.94)	
Other Hispanic	1,037 (4.11)	143 (5.07)	
Other Race	501 (5.11)	49 (5.14)	
Educational status (%)			< 0.0001
Less than high school	1,449 (5.54)	190 (7.71)	
High school	4,903 (35.70)	659 (45.69)	
More than high school	6,094 (58.76)	553 (46.60)	
Diabetes mellitus (%)			0.20
No	10,210 (87.32)	1,180 (88.85)	
Yes	2,245 (12.68)	225 (11.15)	
Hypertension (%)			0.02
No	7,144 (62.48)	869 (66.29)	
Yes	5,309 (37.52)	536 (33.71)	
Depression (%)			< 0.0001
No	11,456 (93.20)	1,190 (84.24)	
Yes	985 (6.80)	212 (15.76)	
Recreational activity (%)			< 0.0001
No	7,331 (53.09)	910 (61.27)	
Yes	5,124 (46.91)	495 (38.73)	
Work activity (%)			< 0.001
No	6,411 (45.03)	823 (52.44)	
Yes	6,044 (54.97)	582 (47.56)	
Active commuting (%)			0.89
No	9,279 (75.05)	1,049 (74.83)	
Yes	3,176 (24.95)	356 (25.17)	
Smoke (%)			0.31
No	9,673 (77.16)	1,092 (75.44)	
Yes	2,779 (22.84)	313 (24.56)	
Drinking (%)			< 0.0001
No	4,002 (26.27)	564 (33.38)	
Yes	8,436 (73.73)	840 (66.62)	

PIR, Family income to poverty ratio.

### 3.2 Relationships between individual vegetable types and constipation

Multivariate analyses were next conducted to explore the relationships between particular vegetable types and constipation status (Table 2). Total vegetable intake and constipation were negatively correlated under the unadjusted model (OR: 0.85, 95%CI: 0.81–0.88), and this same relationship was evident for the intake of non-starchy vegetables, including dark green, total red and orange, and other vegetables, although this relationship was relatively weak with respect to the intake of total starchy vegetables. The ORs for total red and orange vegetable intake (OR: 0.68, 95%CI: 0.60–0.77) were lower than those for the intake of dark green vegetables (OR: 0.76, 95%CI: 0.64–0.90). Comparable relationships were detected under Model 1, revealing that tomatoes exhibited the lowest OR (OR: 0.66, 95%CI: 0.59–0.76), while potato intake was not significantly related to constipation (*p* > 0.05). This remained true when using Model 2 (adjusted for age, sex, ethnicity) and Model 3 (further adjusted for education, BMI, energy intake, total fat intake, total carbohydrate intake, total fruit intake, legumes intake, smoking, drinking, hypertension, depression, recreational activity, work activity, active commuting, and diabetes mellitus), with dark green vegetables and tomatoes exhibiting the strongest ORs under the fully adjusted Model 3 (dark green OR: 0.81, 95%CI: 0.66–0.99, *p* = 0.04; tomato OR: 0.80, 95%CI: 0.71–0.91, *p* = 0.001). In contrast, none of the starchy vegetable subtypes were significantly associated with constipation status under Models 2 or 3.

**Table 2:** Logistic regression analyses of the relationships between particular vegetable types and constipation status.

	Model 1		Model 2		Model 3	
	OR (95% CI)	*p* value	OR (95% CI)	*p* value	OR (95% CI)	*p* value
Total vegetable (cup eq.)	0.85 (0.81,0.88)	<0.0001	0.88 (0.84,0.92)	<0.0001	0.92 (0.87,0.96)	0.002
Dark green vegetable (cup eq.)	0.76 (0.64,0.90)	0.003	0.73 (0.60,0.88)	0.002	0.81 (0.66,0.99)	0.04
Total red and orange vegetable (cup eq.)	0.68 (0.60,0.77)	<0.0001	0.73 (0.65,0.83)	<0.0001	0.80 (0.70,0.91)	0.002
Tomato (cup eq.)	0.66 (0.59,0.76)	<0.0001	0.74 (0.66,0.83)	<0.0001	0.80 (0.71,0.91)	0.001
Other red and orange vegetable (cup eq.)	0.66 (0.46,0.96)	0.03	0.67 (0.46,0.99)	0.05	0.79 (0.54,1.16)	0.22
Total starchy vegetable (cup eq.)	0.92 (0.85,1.00)	0.06	1.00 (0.92,1.09)	0.95	1.01 (0.92,1.11)	0.80
Potato (cup eq.)	0.93 (0.85,1.02)	0.12	1.02 (0.92,1.12)	0.70	1.03 (0.92,1.15)	0.61
Other starchy vegetable (cup eq.)	0.87 (0.72,1.06)	0.17	0.92 (0.76,1.12)	0.41	0.94 (0.77,1.14)	0.49
Other vegetable (cup eq.)	0.78 (0.72,0.84)	<0.0001	0.81 (0.75,0.87)	<0.0001	0.88 (0.81,0.95)	0.003

Model 1 had no covariate-adjusted; Model 2 adjusted age, sex, and ethnicity; Model 3 adjusted age, gender, ethnicity, education, BMI, energy intake, total fat intake, total carbohydrate intake, total fruit intake, legumes intake, smoking, drinking, hypertension, depression, recreational activity, work activity, active commuting, and diabetes mellitus.

The relationship between individual vegetable components and constipation status was additionally examined when utilizing different approaches to defining constipation. When defining constipation based on stool consistency, every one unity increase in the intake of tomatoes was related to a 17% reduction in the risk of constipation under the fully adjusted model (OR: 0.83, 95%CI: 0.71–0.97), whereas potato or total starchy vegetable were unrelated to constipation (Supplementary Table S1). When defining constipation according to stool frequency, similar results were observed in Model 1 and 2 such that tomato intake was associated with constipation, while potato or total starchy vegetable intake was not related to constipation (Supplementary Table S2).

**Figure 1:**
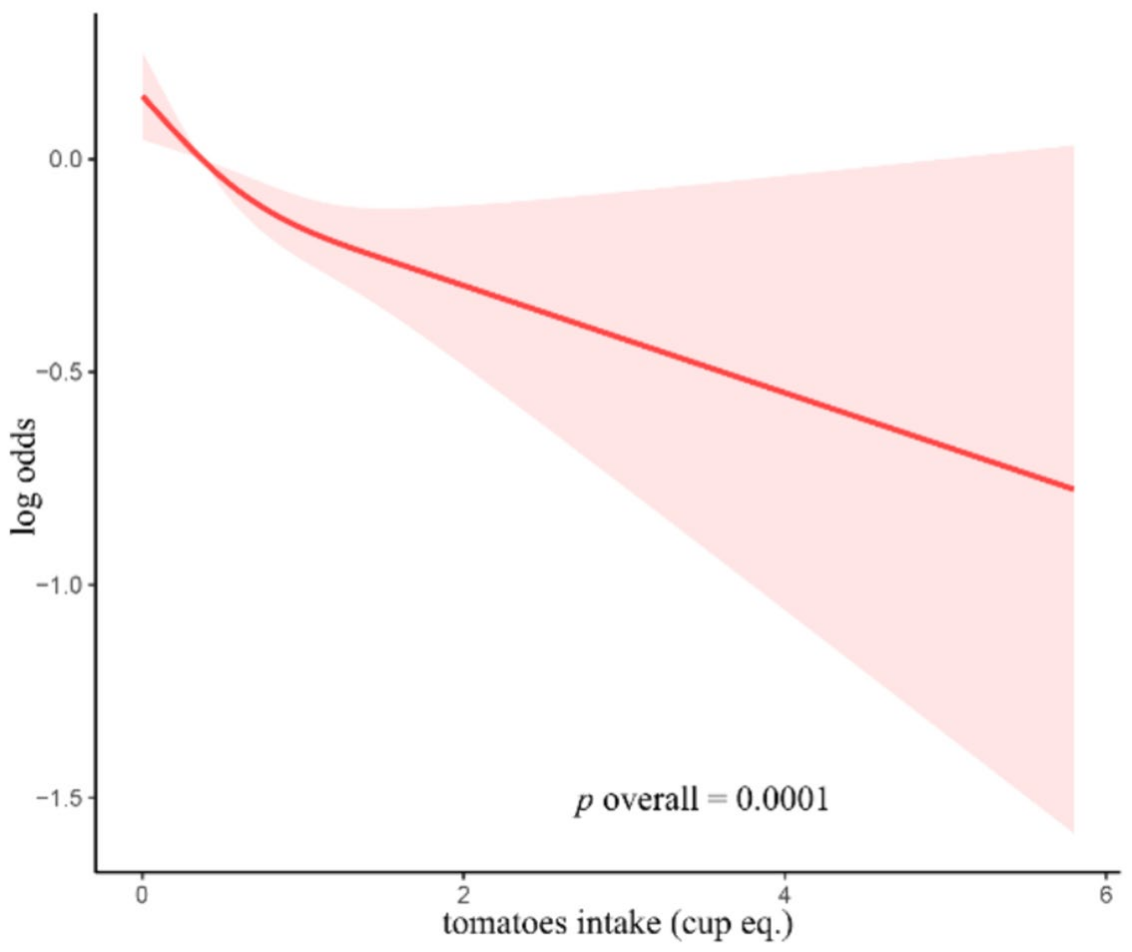
RCS curves for the associations between the consumption of tomatoes and the occurrence of constipation.

As tomato intake exhibited strong predictive performance in all three models, it was included in subsequent RCS and subgroup analyses. RCS curves indicated that tomato intake was negatively correlated with constipation incidence in a nearly linear manner (Figure 1).

### 3.3 Subgroup analyses

To clarify the degree to which the relationships between the intake of tomatoes and the rate of constipation were robust in particular demographic groups of patients, subgroup analyses were performed (Table 3). Consistent results were observed in all subgroups, with particularly significant interaction *p* values when subgroup analyses were performed based on ethnicity (*p* < 0.001).

**Table 3:** Subgroup analyses focused on the relationship between the intake of tomatoes and the incidence of constipation.

	OR (95% CI)	*p* value	*p* for interaction
Sex			0.67
Female	0.74 (0.64,0.87)	<0.001	
Male	0.69 (0.54,0.90)	0.01	
Race			< 0.001
Mexican American	0.90 (0.68,1.19)	0.45	
Non-Hispanic Black	0.96 (0.76,1.22)	0.75	
Non-Hispanic White	0.64 (0.54,0.76)	<0.0001	
Other Hispanic	0.89 (0.62,1.27)	0.51	
Other Race	0.16 (0.05,0.49)	0.002	
Educational status			0.13
Less than high school	0.78 (0.50,1.19)	0.24	
High school	0.79 (0.65,0.96)	0.02	
More than high school	0.61 (0.50,0.75)	<0.0001	
Diabetes mellitus			0.96
No	0.67 (0.58,0.77)	<0.0001	
Yes	0.68 (0.46,1.00)	0.05	
Hypertension			0.39
No	0.64 (0.54,0.76)	<0.0001	
Yes	0.73 (0.58,0.92)	0.01	
Depression			0.08
No	0.72 (0.63,0.82)	<0.0001	
Yes	0.53 (0.37,0.75)	<0.001	
Recreational activity			0.06
No	0.62 (0.54,0.73)	<0.0001	
Yes	0.77 (0.64,0.92)	0.01	
Work activity (%)			0.45
No	0.72 (0.59,0.87)	0.002	
Yes	0.64 (0.52,0.78)	<0.0001	
Active commuting (%)			0.44
No	0.69 (0.60,0.80)	<0.0001	
Yes	0.61 (0.46,0.82)	0.002	
Smoke			0.37
No	0.70 (0.59,0.82)	<0.0001	
Yes	0.60 (0.46,0.78)	<0.001	
Drinking			0.56
No	0.73 (0.59,0.90)	0.004	
Yes	0.67 (0.56,0.80)	<0.0001	

## 4 Discussion

The present study was a large observational analysis of adults in the United States, which revealed that the intake of non-starchy dark green, red/orange, and other vegetables was associated with lower constipation risk, whereas the same was not true for starchy vegetables or potatoes. Changing the criteria used to define constipation did not impact these results. Of all analyzed vegetable subtypes, tomatoes were the most closely associated with lower rates of constipation. In subgroup analyses, a stable link between tomato intake and constipation was observed in various populations, with significant interaction *p* values being detected as a function of ethnicity.

Notably, several prior studies have examined the association between overall vegetable intake or other dietary factors and constipation without specifically examining the effects of specific vegetable subtypes in this setting. According to the most recent Healthy Eating Index (2015) published by the Dietary Guidelines for Americans team determined that higher total vegetable intake was related to lower constipation risk (14). Stricter adherence to a Mediterranean diet rich in vegetables has similarly been tied to lower rates of constipation (13). An epidemiological analysis performed in China also determined that lower constipation rates were related to the consumption of more vegetables (4). This may be because the high soluble fiber content in many vegetables can improve the influx of water, softening stools and adding additional bulk, thus reducing the odds of constipation (6, 18). Vegetables can also introduce bacteria that may beneficially affect the microbiota in the host, contributing to greater functional diversity and more positive outcomes (19). While the findings of these past studies align well with the present data in terms of the negative correlation between the intake of vegetables and constipation risk, there remains a pressing lack of data with respect to how different vegetable types impact this risk.

Here, the relationship between four vegetable classes (dark green, red and orange, starchy, and other) and constipation was assessed in accordance with United States Dietary Guidelines. Separate analyses were also conducted with a focus on participant intake of tomatoes and potatoes, as subtypes of red/orange and starchy vegetables, respectively. In Luxembourg, an analysis of 1,431 adults determined that the intake of potatoes and other starchy vegetables was related to lower rates of constipation (15). Potatoes are rich in fiber, vitamin C, vitamin B6, iron, potassium, folate, and other nutrients, in addition to being widely consumed in much of the world (20). However, no link between the intake of potatoes or other starchy vegetables and constipation rates was herein detected. This may be attributable to the fact that potatoes are often present in unhealthy Western diets in the form of foods such as French fries, mashed potatoes, and potato chips (21). Janett et al. also found that proinflammatory diets, including potatoes, are associated with higher circulating IL-6 and CRP levels (22). As these diets exhibit greater inflammatory potential, they may have an effect on the gastrointestinal microbiome, in turn contributing to greater constipation risk (19, 23).

Non-starchy vegetable intake was closely associated with constipation risk in this study population. While the utilization of varying definitions of constipation have the potential to result in some inconsistencies or biases with respect to study findings (24), the close relationship between non-starchy vegetables and constipation risk remained intact whether constipation was defined based upon stool frequency or stool consistency, emphasizing that these findings are robust. Of all analyzed forms of vegetable intake, the consumption of tomatoes was most strongly associated with constipation. There are several potential explanations for this finding. For one, tomatoes represent a rich source of lycopene, which has the potential to suppress oxidative stress and to alleviate inflammation in the intestines. Indeed, in animal model systems, lycopene administration has been shown to both induce the production of anti-inflammatory cytokines (IL-10, TGF-β) while also suppressing the release of pro-inflammatory mediators (IL-6, IL-8, IL-1β, and TNF-α) (25, 26). It is also possible that lycopene regulates tight junction protein expression, thereby preserving the integrity of the intestinal epithelial (27). In a murine dextran sodium sulfate (DSS)-induced colitis model, lycopene is also capable of altering the composition of the gut microbiome by increasing relative Bifidobacterium and Lactobacillus abundance while contributing to lower Proteobacteria levels (28). Lycopene may thus represent an important tomato derivative conducive to better intestinal health and the alleviation of constipation owing to its ability to suppress oxidative stress, preserve the function of the intestinal mucosal epithelial barrier function (29), inhibit inflammation, and restore the homeostasis of the gut flora (30, 31).

Both age and sex have been identified as key constipation-related risk factors (32). The incidence of constipation in the present study cohort was over twice as high among women as compared to men, although age was not detected as a relevant risk factor in this case, in line with prior NHANES data (33). Subgroup analyses indicated that constipation risk was higher among individuals who did not engage in recreational activity and individuals with depression, also supporting past research findings. Efforts to improve physical activity and to alleviate depression thus represent viable modifications with the potential to help reduce constipation risk. A significant interaction between tomatoes and constipation risk as a function of ethnicity may be tied to dietary and lifestyle variations.

This study has a number of notable strengths. As these findings were based on NHANES datasets, which comprise a nationally representative, large-scale sample population, these results are highly reliable. In addition, this is the first published report specifically focusing on the effects of different vegetable types on constipation. However, there are some limitations to these analyses that warrant acknowledgment. For one, the causal nature of these associations could not be assessed owing to the fact that these were cross-sectional data. Secondly, the definition of constipation in this study failed to satisfy the diagnostic criteria for functional constipation according to Rome IV Criteria. In addition, the fact that dietary data were obtained through 24-h recall interviews means that they are inevitably susceptible to the potential for recall bias. Moreover, the recall time interval was 24 h, meaning that the long-term dietary pattern of participants were not included in the database. Furthermore, due to limited access to complete data, family history of constipation, hypothyroidism, celiac disease, and duration of constipation were not included for further analysis in this study. Lastly, despite efforts to control for covariates throughout, this study did not consider other potential confounding variables that may have influenced the results of these analyses.

## 5 Conclusion

In summary, the present results suggest that consuming higher amounts of non-starchy dark green, red and orange, and other vegetables can reduce the potential for the incidence of constipation. In contrast, potato or total starchy vegetable intake was not related to such constipation risk. As a specific subtype of red and orange vegetables, tomatoes were even more strongly associated with decreased constipation risk. Together, these data offer an opportunity for more effective dietary modification in an effort to prevent or control constipation.

## Data availability statement

Publicly available datasets were analyzed in this study. This data can be found here: https://www.cdc.gov/nchs/nhanes/index.htm.

## Ethics statement

The studies involving humans were approved by the National Center for Health Statistics Ethics Review Board. The studies were conducted in accordance with the local legislation and institutional requirements. The participants provided their written informed consent to participate in this study.

## Author contributions

CJ: Conceptualization, Data curation, Formal analysis, Funding acquisition, Investigation, Methodology, Project administration, Resources, Software, Supervision, Validation, Visualization, Writing – original draft, Writing – review & editing. YS: Conceptualization, Data curation, Formal analysis, Funding acquisition, Investigation, Methodology, Project administration, Resources, Software, Supervision, Validation, Visualization, Writing – original draft, Writing – review & editing.
