# Supplementary material for: The effects of specific vegetable subtypes on constipation incidence in the general United States population
Source: Front Nutr. 2024 Jul 24;11:1403636. doi: 10.3389/fnut.2024.1403636 (PMC11304535; doi:10.3389/fnut.2024.1403636)
Supplement: Supplementary file 1 [file Table_1.docx]

Supplementary Table S1: Association between particular vegetable types and constipation defined by stool consistency.

|  | Model 1 | | Model 2 | | Model 3 | |
| --- | --- | --- | --- | --- | --- | --- |
|  | OR (95% CI) | *p* value | OR (95% CI) | *p* value | OR (95% CI) | *p* value |
| Total vegetable (cup eq.) | 0.86(0.83,0.90) | <0.0001 | 0.89(0.85,0.93) | <0.0001 | 0.92(0.88,0.97) | 0.002 |
| Dark green vegetable (cup eq.) | 0.80(0.67,0.95) | 0.01 | 0.77(0.64,0.93) | 0.01 | 0.85(0.70,1.02) | 0.08 |
| Total red and orange vegetable (cup eq.) | 0.72(0.63,0.82) | <0.0001 | 0.77(0.68,0.88) | <0.001 | 0.83(0.72,0.95) | 0.01 |
| Tomato (cup eq.) | 0.72(0.61,0.83) | <0.0001 | 0.78(0.67,0.90) | 0.002 | 0.83(0.71,0.97) | 0.02 |
| Other red and orange vegetable (cup eq.) | 0.72(0.52,0.98) | 0.04 | 0.72(0.52,0.99) | 0.04 | 0.80(0.58,1.11) | 0.17 |
| Total starchy vegetable (cup eq.) | 0.91(0.84,0.99) | 0.03 | 0.98(0.90,1.07) | 0.64 | 0.99(0.90,1.09) | 0.79 |
| Potato (cup eq.) | 0.92(0.83,1.02) | 0.09 | 0.99(0.90,1.10) | 0.92 | 1.00(0.89,1.13) | 0.94 |
| Other starchy vegetable (cup eq.) | 0.87(0.72,1.05) | 0.15 | 0.90(0.74,1.10) | 0.29 | 0.91(0.75,1.11) | 0.33 |
| Other vegetable (cup eq.) | 0.81(0.74,0.88) | <0.0001 | 0.83(0.76,0.90) | <0.0001 | 0.89(0.81,0.97) | 0.01 |

Model 1 had no covariate adjusted.

Model 2 adjusted age, sex, and ethnicity.

Model 3 adjusted age, gender, ethnicity, education, BMI, energy intake, smoking, drinking, hypertension, depression, recreational activity, and diabetes mellitus.

Supplementary Table S2: Association between particular vegetable types and constipation defined by stool frequency.

|  | Model 1 | | Model 2 | | Model 3 | |
| --- | --- | --- | --- | --- | --- | --- |
|  | OR (95% CI) | *p* value | OR (95% CI) | *p* value | OR (95% CI) | *p* value |
| Total vegetable (cup eq.) | 0.80(0.73,0.87) | <0.0001 | 0.84(0.77,0.91) | <0.001 | 0.90(0.82,0.99) | 0.03 |
| Dark green vegetable (cup eq.) | 0.61(0.43,0.87) | 0.01 | 0.57(0.38,0.83) | 0.005 | 0.67(0.46,0.98) | 0.04 |
| Total red and orange vegetable (cup eq.) | 0.56(0.40,0.77) | <0.001 | 0.63(0.46,0.87) | 0.01 | 0.73(0.52,1.02) | 0.06 |
| Tomato (cup eq.) | 0.59(0.45,0.77) | <0.001 | 0.68(0.52,0.89) | 0.01 | 0.77(0.58,1.01) | 0.06 |
| Other red and orange vegetable (cup eq.) | 0.41(0.16,1.11) | 0.08 | 0.43(0.16,1.20) | 0.10 | 0.59(0.22,1.62) | 0.29 |
| Total starchy vegetable (cup eq.) | 0.93(0.80,1.08) | 0.34 | 1.03(0.87,1.22) | 0.70 | 1.06(0.90,1.24) | 0.49 |
| Potato (cup eq.) | 0.93(0.79,1.11) | 0.44 | 1.05(0.87,1.27) | 0.62 | 1.07(0.88,1.29) | 0.48 |
| Other starchy vegetable (cup eq.) | 0.87(0.64,1.20) | 0.40 | 0.96(0.70,1.32) | 0.81 | 1.02(0.75,1.37) | 0.91 |
| Other vegetable (cup eq.) | 0.71(0.62,0.81) | <0.0001 | 0.75(0.66,0.86) | <0.0001 | 0.85(0.74,0.98) | 0.03 |

Model 1 had no covariate adjusted.

Model 2 adjusted age, sex, and ethnicity.

Model 3 adjusted age, gender, ethnicity, education, BMI, energy intake, smoking, drinking, hypertension, depression, recreational activity, and diabetes mellitus.
